# Immigrant, Refugee, and Indigenous Canadians’ Experiences With Virtual Health Care Services: Rapid Review

**DOI:** 10.2196/47288

**Published:** 2023-10-09

**Authors:** Megan MacPherson

**Affiliations:** 1 Virtual Health Fraser Health Authority Surrey, BC Canada

**Keywords:** delivery of health care, emigrants and immigrants, health disparate, indigenous Canadians, minority and vulnerable populations, refugees, telemedicine

## Abstract

**Background:**

The remote, dispersed, and multicultural population of Canada presents unique challenges for health care services. Currently, virtual care solutions are being offered as an innovative solution to improve access to care.

**Objective:**

Given the inequities in health care access faced by immigrant, refugee, and Indigenous Canadians, this review aimed to summarize information obtained from original research regarding these people’s experiences with virtual care services in Canada.

**Methods:**

We conducted a rapid review following published recommendations. MEDLINE and CINAHL were searched for studies relating to virtual care and Canadian immigrants, refugees, or Indigenous peoples. Peer-reviewed articles of any type were included so long as they included information on the experiences of virtual care service delivery in Canada among the abovementioned groups.

**Results:**

This review demonstrates an extreme paucity of evidence examining the experiences of immigrant, refugee, and Indigenous groups with virtual care in Canada. Of the 694 publications screened, 8 were included in this review. A total of 2 studies focused on immigrants and refugees in Canada, with the remaining studies focusing on Indigenous communities. Results demonstrate that virtual care is generally accepted within these communities; however, cultural appropriateness or safety and inequitable access to wireless services in certain communities were among the most cited barriers.

**Conclusions:**

Little evidence exists outlining immigrants’, refugees’, and Indigenous peoples’ perspectives on the landscape of virtual care in Canada. The development of virtual care programming should take into consideration the barriers, facilitators, and recommendations outlined in this review to improve equitable access. Further, developers should consult with local community members to ensure the appropriateness of services for immigrant, refugee, and Indigenous communities in Canada.

## Introduction

In 2021, roughly 1 in 3 Canadians identified as Indigenous or were part of a racialized group [1]. This includes 8.3 million landed immigrants or permanent residents, nearly a quarter million new refugees admitted as permanent residents, and 1.8 million Indigenous people according to the 2021 Canadian census [2]. These populations are defined as “equity-owed” groups within Canada because of the significant health disparities they face likely due to unequal access, opportunities, and resources provided to them, resulting in inaccessible and poorer quality health care services [3-6]. For example, 1 study assessing the self-reported health of Canadian immigrants found a rapid decline in overall health during the initial 2 years following settlement in Canada, with significantly lower self-reported health among non-European immigrants (Arab African, West Asian, South Asian, and Chinese groups) [7]. It has been suggested that these health disparities are likely due to social determinants of health (including poverty, food insecurity, and a lack of employment opportunities) and postimmigration experiences of discrimination [7,8].

Similarly, Indigenous peoples in Canada experience significant health disparities, such as a higher incidence of chronic diseases and disability [9,10]. Such disparities are likely a result of social determinants of health as well as a lack of access to adequate and culturally appropriate health care [11,12], and experiences of racism and social exclusion [13]. In a qualitative study conducted within British Columbia [6], urban Indigenous peoples noted that time was a major barrier to accessing care. Specifically, they experienced delays in seeing medical professionals, receiving diagnoses, and receiving appropriate treatment. Participants also mentioned that both the limited time during appointments to discuss their health concerns and the long wait times impeded their ability to access care [6]. Further, Indigenous communities are among the most geographically remote within Canada, with roughly 60% of Indigenous peoples living in predominantly rural areas [14]. The proximity of Indigenous peoples to major health care centers results in additional barriers in terms of transportation and physical access to specialty services [15].

Similarly, Canadian immigrants and refugees have noted barriers to accessing quality care in relation to culture, communication, socioeconomic status, and health care system structure [4,5,16]. For example, a significant number of Canadian immigrants and refugees are unable to converse with health care providers due to language barriers that impact both access to and quality of care [17-23]. Further, many Canadian immigrants have described not having the time or resources to attend medical appointments [23-25]. Given the inequities and disparities faced by immigrant, refugee, and Indigenous communities within Canada, innovative care strategies may enhance access to care and improve health outcomes among these groups.

Over the last several decades, there have been numerous efforts to implement virtual care (ie, the delivery of health care services and information through remote technologies) to improve access, quality, and safe health care delivery for Canadians [26]. Virtual care (when designed in a user-centered and equity-focused manner) has the potential to alleviate barriers faced by equity-owed groups in Canada. Specifically, virtual care can increase accessibility to specialized services, reduce travel time, and shorten time away from home and work [27] and has been identified as a key mechanism for improving access to health care services [28]. To improve the equitable design and implementation of virtual care services within Canada, this review aims to synthesize evidence pertaining to the contextual advantages or disadvantages faced by immigrant, refugee, and Indigenous Canadians when using virtual care services.

## Methods

### Overview

This rapid review was conducted following published recommendations [29] and aimed to answer the following questions: (1) what advantages or disadvantages are commonly experienced by equity-owed groups when accessing and engaging with virtual care? (2) What strategies are suggested to improve the equitable uptake of virtual care services in Canada? To improve methodological rigor, the PRISMA-ScR (Preferred Reporting Items for Systematic Reviews and Meta-Analyses Extension for Scoping Reviews) checklist [30] was also used to guide this review (Multimedia Appendix 1).

### Search Strategy and Data Sources

Database-specific searches were developed for MEDLINE and CINAHL. The search strategy probed 4 different areas of inquiry: virtual care, immigrants and refugees, Indigenous peoples, and Canada. The full search strategy can be found in Multimedia Appendix 2.

Titles and abstracts were searched in the databases MEDLINE (EBSCO) and CINAHL (EBSCO) from inception to December 2022. As the objective was to obtain an expedited overview of current research, reference lists of the included studies and gray literature were not explored.

### Eligibility Criteria

Publications were retained for review if they met the following criteria: (1) written in English; (2) a peer-reviewed article; (3) concerned immigrant, refugee, or Indigenous populations living in Canada; and (4) reported on opinions or experiences of virtual care services of any type. No limits were placed on study design, type of virtual care service, or publication date.

### Study Selection

All identified studies were imported into the systematic review software Covidence (Veritas Health Innovation Ltd), where duplicate studies were automatically removed. A single reviewer screened titles and abstracts, then full texts, for inclusion.

### Data Extraction and Synthesis

Once all articles were screened, a custom data extraction form was developed within Covidence. A single reviewer extracted data broadly pertaining to the population and their opinions or experiences with virtual care services. Results were then narratively summarized to provide an organized portrait of the data and group results into related themes.

## Results

### Overview

**Figure 1:**
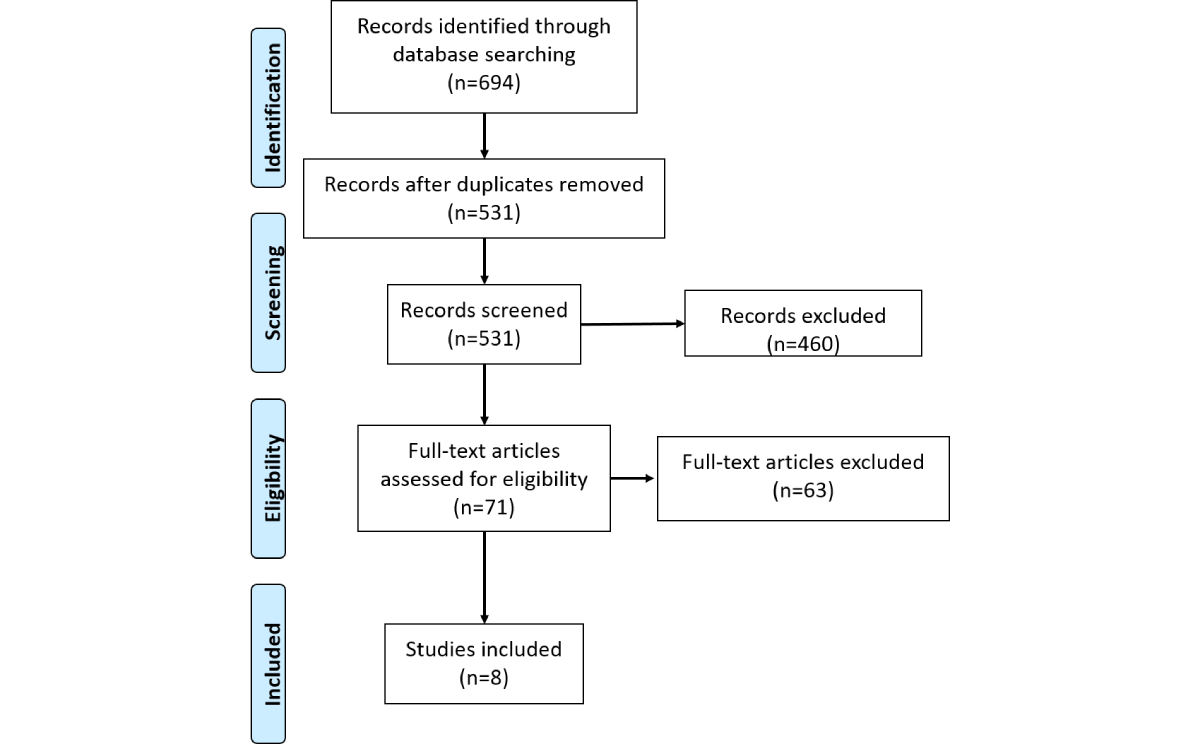
PRISMA (Preferred Reporting Items for Systematic Reviews and Meta-Analyses) flow diagram.

The initial search yielded 694 results, of which 8 met the eligibility criteria and were included in data extraction [31-38] (Figure 1). A total of 2 studies focused on immigrants and refugees in Canada [32,37]. Specifically, refugees within the Hynie et al [37] study were primarily from Syria, Eritrea, Iran, Ethiopia, Columbia, and Somalia, and immigrants and refugees within Cortinois et al’s [32] study were primarily from Mexico, Columbia, and Ecuador. The remaining studies focused on Canadian Indigenous communities [31,33-36,38]. The included studies comprised review articles [33,34,38], focus groups or interviews [31,32,37], and 1 cohort study with a mixed methods design [35]. Specific virtual care services included call centers providing health-related information to immigrant populations [32], SMS text messaging for Indigenous youths who use illicit drugs [35], assistive technologies for aging in place [34], and more broad virtual care services for individuals with diabetes [33], chronic pain [36], and mental health conditions [31,37].

### Experiences With Virtual Care

#### Overview

**Figure 2:**
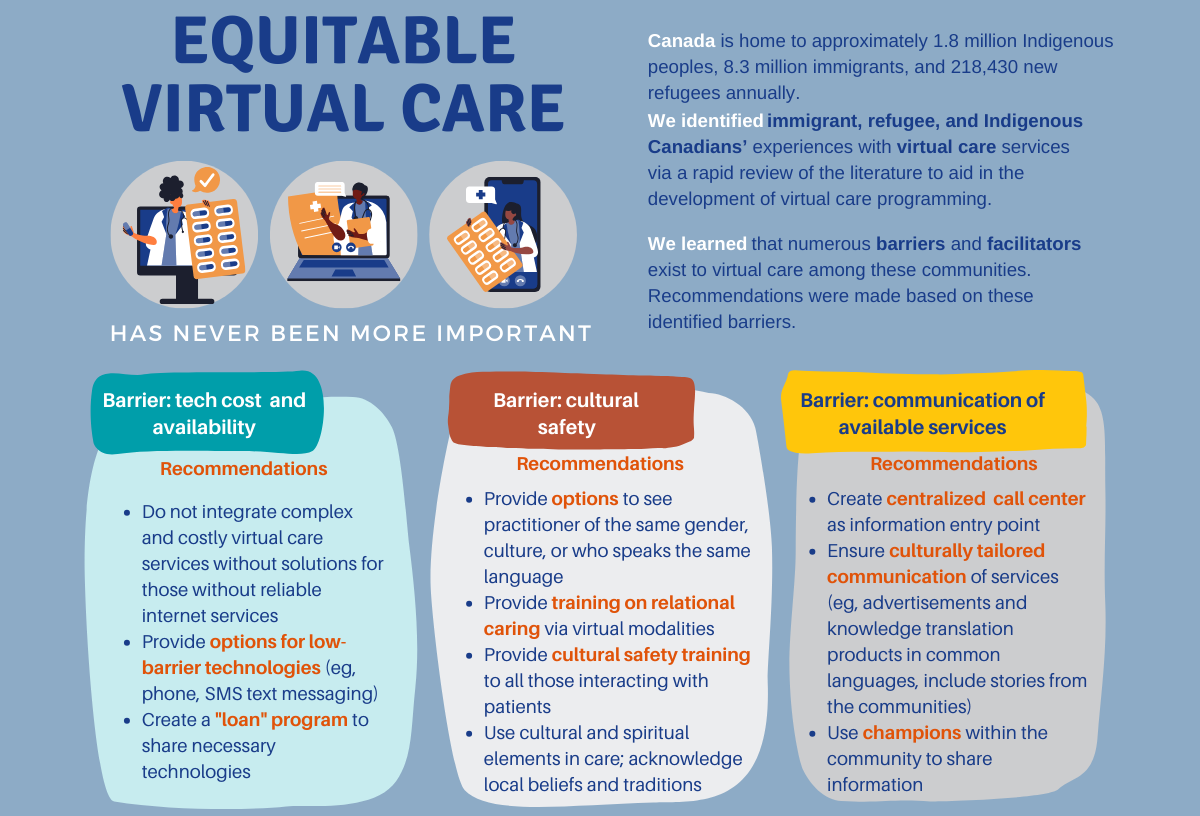
Summary of key recommendations.

Within the included studies, virtual care was consistently identified as an acceptable mode of health care delivery by equity-owed groups; however, several advantages and disadvantages were noted, and recommendations were made to improve equitable virtual care. A total of 3 broad themes were identified regarding the advantages and disadvantages of accessing and engaging with virtual care: cultural appropriateness and safety [31-38], access and skills [31,34-38], and information about available services [32,37]. See Textbox 1 for a comprehensive list of advantages and disadvantages and Textbox 2 and Figure 2 for suggestions to improve virtual care service provision.

**Advantages and disadvantages of virtual care service provision.**

**Advantages**

- Cultural appropriateness and safety
- Distance may facilitate dialogue and openness in some people who feel more comfortable being physically distant from the clinician [31]
- Use of Indigenous health workers [33,36]
- Virtual care can help create a space where individuals can communicate, share, and heal in their own language [31,32,37]
- Goal for virtual care to enhancing interdependence, not independence [34,35]
- Access
- Increased access to services [31,35]. Decreased health care cost [31,36-38] and travel time [36-38]
- Convenient access to educational materials [38]
- Information about available services
- Informed community network to share information about available services [37]

**Disadvantages**

- Cultural appropriateness and safety
- Some may find that distance can detract from the therapeutic relationship [31]. People cannot rely on nonverbal or body language [37]
- Cameras can make some people uncomfortable (specifically in group settings where people may want to remain anonymous) [37]
- Culturally inappropriate and unsafe virtual care (eg, not acknowledging one’s identity; failing to provide culturally relevant resources, stereotyping and bias, lack of cultural competence, language barriers) [33,34,37,38]
- Access
- Inequitable access to internet services [31,34,36]
- Technology cost [34,35,37]
- Lack of digital content specific to the cultures and languages of communities [34]
- Problems with technology [31,37]
- Privacy concerns [31,38]
- Information about available services
- Lack of widespread advertising of virtual care services [32,37]
- Disjointed, low-quality information sources (eg, word of mouth, printed materials, the radio, television, and the internet with inconsistent messaging) [32]

**Recommendations and suggestions to improve virtual care service provision.**

**Recommendations to improve cultural appropriateness and safety**

- Clinician training on how to provide relational caring through virtual care [37]
- Offer a first meeting in person before moving on the web [31,37]
- Provide choices for group education in which patients can remain anonymous and participate through the chat function or with their video off
- Use cultural and spiritual elements, acknowledge local beliefs and traditions [33,34], and include family and community [34]
- Use interpreters or consult Indigenous health workers and adopt a holistic perspective of health [38]
- Consider the following questions when developing virtual care services [34]: is the content relevant to the community? Is the illness of noted concern to the community? Are suggestions for prevention and management realistic given geographic location and socioeconomic status (eg, in one study participants noted that if people do not have running water in their homes, how helpful is telemental health [31])?

**Recommendations to improve access**

- Provide options for low-barrier technologies (eg, phone call and SMS text messaging)
- Create a “loan” program that provides patients with necessary technologies
- Provide written recommendations following appointment to be referred back to
- Provide options to connect with same sex, same culture, and same language clinicians
- Ensure that digital content is available in all languages common to the community
- Provide technical support to both health care providers and recipients [36]

**Recommendations to improve information about available services**

- Ensure that communities are aware of existing virtual care services [37]
- Promote services on websites, social media, build searchable databases of available services for both health care providers and recipients to facilitate access to information [37]
- Have brochures available in community health centers and create short videos about virtual care services featuring community members [31]

#### Cultural Appropriateness and Safety

The most cited challenge noted in this review was the cultural appropriateness and cultural safety of virtual care interventions [33,34,37,38]. Culturally safe virtual care refers to the provision of health care services through digital platforms in a manner that respects and addresses the cultural needs, values, and preferences of individuals or communities. It recognizes the importance of cultural diversity and seeks to create an inclusive and equitable health care environment that supports the well-being of all patients, regardless of their cultural background. Conversely, when virtual care services are delivered in a culturally safe manner, this can help to create a space where individuals are able to communicate, share, and heal in their own language and in their own environment [31,32,36,37]. Culturally safe health care delivery may also improve engagement with the health care system. For example, a recent systematic review assessing barriers and enablers for Indigenous communities found an increase in both patient satisfaction and attendance (from 20% to 80%) following the implementation of cultural and spiritual services (such as hosting ceremonies under the guidance of spiritual leaders in the community, conducting “Smudge” ceremonies, etc) within telediabetes care [33].

#### Access and Skills

The provision of virtual care can increase access by providing support to broader geographic communities who may not have access to specialists [31,35], increase the ability for individuals to engage with health care providers who speak the same language as them [37], and can potentially decrease the time and cost associated with traveling to health care appointments [31,36-38]. For example, Canadian immigrants and refugees noted that they were spared the time, cost, and inconvenience of traveling long distances for appointments when offered virtual care, which was particularly advantageous for those with young children [37]. Immigrants, refugees, and health care providers additionally noted that virtual care services allowed for more frequent check-ins and greater flexibility, which enabled patients to include important advocates and community members in their appointments [37].

On the other hand, the use of technology within health care has been criticized for increasing the digital divide, in which some groups (eg, immigrants, refugees, and Indigenous Canadians) have inequitable access to internet services [31,34,36], or cannot afford the technologies required for virtual care services [34,35,37]. Further, there is a noted lack of web-based health content tailored to specific cultures or languages for those already marginalized communities, which decreases the usability of available web-based health resources for certain groups [34]. Beyond access to the specific technologies, both health care providers and recipients must also have the necessary skills to engage with the required technologies, highlighting the importance of effective training programs to improve the digital literacy of patients and providers [33,34].

#### Information About Available Services

An important aspect of virtual care services is the ease with which they can be identified and reached, as virtual care is only effective if services reach the communities they serve [37]. For example, newcomers to Canada tend to rely on both formal and informal networks (including friends, sponsors, health, and social providers) to identify available mental health services [37]. Similarly, a study assessing the use of an information call center in Ontario among immigrants noted that, when looking for information, almost all participants were helped by someone they met by accident [32]. This highlights the importance of increasing community knowledge of virtual care services.

## Discussion

### Principal Results

Despite Canada’s universal health care system, access to necessary health care services varies considerably based on factors such as socioeconomic status, ethnicity, location, and culture, among others. Specifically, many immigrant, refugee, and Indigenous Canadians have experienced discrimination within the Canadian health care system [12,39,40]. With more than 10 million Canadians classified within these equity-owed groups [2], inclusive and equitable health care development has the potential to drastically improve the provision of new health care services. This rapid review highlighted several advantages and disadvantages that exist for virtual care programming and service delivery among immigrants, refugees, and Indigenous Canadians. Additionally, recommendations were summarized toward improving the virtual care experience among equity-owed groups in Canada.

The ways in which immigrants, refugees, and Indigenous Canadians navigate the complex health care system in Canada are impacted by multiple factors. The most commonly cited barriers to engaging in virtual care services among these groups are that they are not developed or implemented in a culturally appropriate or safe way, the technologies are not widely available or accessible, and individuals are often unaware of the available services. Some of the key recommendations toward improving virtual care among these equity-owed groups include engaging the community in the development and provision of virtual care services, involving culturally tailored health workers and cultural practices, providing appropriate staff training (to improve the ability to engage with the technology and engage in relational care digitally), and ensuring that available programs are effectively advertised.

### Comparison With Previous Work

The 3 main themes identified in this review (culturally appropriate and safe care, access, and awareness of available services) are well aligned with the existing literature both within and outside of Canada. Numerous reviews have previously been conducted to assess patient experiences with virtual care, primarily in rural settings [41-43] and among older adults [44]. These reviews have identified similar themes to those found in the current review. For instance, while previous reviews do not explicitly discuss cultural appropriateness and safety, a consistent theme revolves around the impact of virtual care on the patient-provider relationship, with both positive and negative implications noted [43-45].

The therapeutic relationship is a crucial aspect of health care and counseling, involving a professional and collaborative alliance between a health care provider and a patient. It focuses on establishing trust, mutual respect, and open communication between the provider and the individual seeking care [46]. The therapeutic relationship creates a safe and supportive environment where patients can express their concerns, emotions, and experiences without judgment. Over time, as patients and health care providers become more familiar with each other, the quality of care improves as providers can deliver more personalized health care interventions, leading to increased patient satisfaction [47].

A strong therapeutic relationship also contributes to more effective patient education, greater trust and patient disclosure, improved patient compliance, and better health outcomes [48,49]. Virtual care has the potential to facilitate the therapeutic relationship [41,43,46]. For example, asynchronous telehealth modalities, such as email, SMS text messaging, and instant messaging, hold promise for building therapeutic relationships due to their widespread use and the convenience they offer in terms of recording, storing, and forwarding digital information without the need for both parties to be present simultaneously [46].

Synchronous modalities, such as telephone and videoconferencing, provide immediate, clear, and real-time communication advantages. Videoconferencing, in particular, is rapidly becoming the primary synchronous modality as it allows for the exchange of both verbal and nonverbal cues during web-based encounters, thereby facilitating many key determinants of normal patient-doctor relationships [46]. Moreover, videoconferencing enables patients to connect with providers in the comfort and convenience of their own communities, reducing the traditional stresses associated with travel for visits and allowing rural patients to focus on their clinical encounters within familiar and supportive environments [43,46]. However, the use of videoconferencing and other forms of virtual care requires patients to have access to devices, the necessary software, and reliable internet connectivity [41]. This raises concerns about technology literacy, particularly among older adults [41,44]. Additionally, rural communities in Canada often face challenges with internet and broadband access, making it difficult to implement video and web-based components in those areas [41,43].

Furthermore, a lack of patient awareness regarding the availability of virtual care services has been identified among rural Canadians [41]. Without understanding the services that are available within their communities, the utility of virtual care will be significantly limited. This highlights the need for improved advertisements and communication between partners in the health care system.

Findings from this review align with previous reviews, highlighting similar themes and challenges faced in the realm of virtual care services. Recognizing these commonalities provides an opportunity to prioritize targeted improvements in order to enhance the therapeutic relationship and maximize the potential benefits of virtual care. By addressing common issues faced by multiple underserved groups such as older adults, rural Canadians, and Indigenous, immigrant, and refugees in Canada, the Canadian health care system can help overcome barriers and create a more inclusive and patient-centered virtual care environment. It is through these prioritized improvements that virtual care services can reach their full potential in meeting the unique needs of diverse populations and ensuring equitable access to quality care.

### Research and Evaluation Implications

With the growing body of literature pertaining to the delivery of virtual care services within Canada [50,51], it is alarming that only 6 articles reported the experiences of Indigenous Canadians and only 2 focused on immigrant or refugee experiences. More research is needed to strengthen the findings of this review and bolster the voices of immigrant, refugee, and Indigenous populations among Canada’s virtual care network. Specifically, immigrants and refugees included in this review were from Syria, Eritrea, Iran, Ethiopia, Columbia, Somalia, Mexico, Columbia, and Ecuador [32,37]. While this does include a large proportion of Canadian refugee populations (the most common countries of birth for new refugees were Syria, Iraq, Eritrea, Afghanistan, and Pakistan), it excludes the large proportion of Canadian immigrants coming from India, the Philippines, and China (~40%) [52]. To generalize the findings from this review to the broader Canadian context, more research is needed to evaluate the experiences of diverse immigrant and refugee groups not captured in this review to garner a more robust understanding of advantages, disadvantages, and recommendations for more equitable virtual care.

Beyond the identification of barriers faced in navigating virtual care systems and service delivery, research is needed on how technologies can be used to facilitate access to care, optimize health, and how to best leverage technologies to decrease the digital divide and improve health equity. Additionally, the articles included in this review focused entirely on immigrant, refugee, and Indigenous Canadians. Further research should explore intersectionality between these populations and other factors such as sex and gender, sexual orientation, and other dimensions that have been noted to influence access to equitable care [53].

In addition to the questions that need to be answered through research, the ways in which research is being conducted should also shift to more inclusive methodologies such as participatory action research, which emphasizes that research must be performed “with” people, not “on” them [54]. The development of new research studies and virtual care interventions should be done “with” community members to ensure that the virtual care services being created are targeting issues identified within the community and that important cultural practices are effectively integrated into the intervention. For example, the Cedar Project [35], a multisite SMS text messaging intervention aimed at reducing HIV vulnerability among Indigenous youths who use illicit drugs, prioritized community partnerships to ensure that they were conducting research in a manner that was culturally safe by creating safe spaces where individual identities, voices, and stories were heard and respected. This was done, in part, by integrating traditional foods and ceremonies into the research process through annual feasts, memorials, and learning Potlatch.

Further, the evaluation of such interventions should be focused on outcomes of importance to the community. While there is a common belief within western medicine that a health intervention is successful only if it increases an individual’s independence, a review of the literature on health-related technologies found that Indigenous users of virtual care services were less concerned with enhancing their independence compared with enhancing interdependence and that users more readily adopted technologies that integrated with their families and communities [34]. It is important to note that perceptions of health may differ substantially across populations, and the success of virtual care interventions is dependent on the integration of these multicultural views into the development and evaluation of virtual care interventions.

### Policy Implications

By focusing future virtual care policy efforts toward equity-owed groups, the quality of virtual care service delivery can improve for all. In fact, the “Quadruple aim,” a widely used framework suggesting that health care systems can be optimized through reducing costs and improving population health, patient experiences, and health care team well-being [55], has recently evolved into the “Quintuple aim” to additionally include health equity (ie, the state in which no one is disadvantaged from achieving their full health potential due to social determinants of health) [56]. To improve health equity, policy makers should set minimum standards [56]. This can be done through the identification of disparities, the design and implementation of interventions aimed to reduce those disparities, investment in equity evaluations, and incentivization to achieve health equity [56]. Findings from this review may be a useful guide to begin the identification of disparities within the Canadian health care context in which to build future equity-focused policies. For example, policies may want to include minimum standards to include non–internet-enabled technologies within virtual care options, as many health care providers have already noted having to adapt their web-based delivery to modalities requiring less bandwidth to accommodate those unable to afford reliable high-speed internet [37].

### Practice Implications

#### Overview

Several areas of focus were identified to better reach and support immigrant, refugee, and Indigenous Canadians when accessing and engaging with virtual care services. Drawing from the reviewed literature, we present the following recommendations (see Textbox 2 for more recommendations based on this review).

#### Language and Culture

There is a need to focus on language and culture within virtual care programs [31,32,37] including the integration of health workers from specific cultures [33]. Culturally tailored health workers who can communicate in the local language may improve cultural safety within care by helping providers better understand the local community. For example, within the context of telediabetes services, the inclusion of Indigenous health workers was a commonly reported enabler [33]. Working collaboratively with the community in this way can help to ensure the adoption of a holistic perspective of health [38], integration of cultural and spiritual elements into care, and acknowledgment and respect for local beliefs and traditions [33,34].

#### Cultural Safety and Relational Care

Health care providers should take additional cultural safety and relational care training to improve their ability to build and maintain a therapeutic relationship in a virtual care context [34]. Delivery of virtual care services may impact the quality of communication between health care providers and patients [31], thereby challenging the creation of a trusting relationship [37]. This challenge is further amplified when working with immigrants and refugees due to language barriers, use of interpreters, cultural differences, and patients’ unfamiliarity and discomfort with western medicine models [37]. On top of those existing barriers to building a trusting alliance with people of a different culture, virtual care services have been noted to decrease feelings of connection between care provider and recipient [31,37]. This may be due to an inability to rely on body language when talking on the phone, distractions in the patient’s home environment during appointments, and a lack of training on how to engage in relational care digitally [37]. Cultural and relational care training should encompass understanding and respecting the cultural values, beliefs, and practices of individuals and their communities. Additionally, by acknowledging and involving family and community members in the health care process, providers can better support the cultural needs and preferences of patients [34].

#### Inclusion of Communities

The planning, development, and delivery of virtual care interventions should include newcomer and Indigenous communities to promote services that are more equitable, useful, and usable [35]. Involving these equity-owed groups throughout the virtual care life cycle empowers individuals and provides them with a sense of agency in their own health care experiences [57].

#### Improved Navigation

Navigation could be improved through the establishment of a central hub or call center [32], as well as web-based materials that provide information in all local languages [34]. A centralized information call center has been reported to be a trusted and helpful source of information by recent immigrants and has the potential to serve as the initial point of contact for recent immigrants if able to reach them early in the resettlement process [32].

These recommendations, although based on a synthesis of multiple articles, call for further research and a broader evidence base. By addressing these areas of focus, health care providers and policy makers can take initial steps toward improving the accessibility, cultural safety, and effectiveness of virtual care services for immigrant, refugee, and Indigenous Canadians.

### Limitations

This rapid review was limited to peer-reviewed publications, was completed by a single reviewer, and did not search the gray literature. As such, it is important to note that this review is not exhaustive, and other relevant evidence likely exists outside of the peer-reviewed scientific literature that could have contributed to the findings and may have been inadvertently excluded. Further, the recommendations made in this review (see Textbox 2 and Figure 2) are based on populations and results from only 8 articles, of which the quality of the included studies was not assessed. Finally, this review was limited to English language articles in the Canadian context; as such, future efforts should expand this investigation to other countries and non-English journals to improve the generalizability of results.

### Conclusions

There are few studies outlining immigrant, refugee, and Indigenous perspectives on the landscape of virtual care in Canada. While virtual care is generally well accepted within these communities, cultural appropriateness, the safety of virtual care, and inequitable access to wireless services in certain communities were among the most cited barriers. Findings from this review may be useful as a guide to planning and implementing new virtual care services that improve care for immigrant, refugee, and Indigenous communities.

## Appendix

Multimedia Appendix 1 PRISMA-ScR Checklist.

Multimedia Appendix 2 Search strategy for MEDLINE and CINAHL.

## Abbreviations

PRISMA-ScR: Preferred Reporting Items for Systematic Reviews and Meta-Analyses Extension for Scoping Reviews
