# Supplementary material for: Immigrant, Refugee, and Indigenous Canadians’ Experiences With Virtual Health Care Services: Rapid Review
Source: JMIR Hum Factors. 2023 Oct 9;10:e47288. doi: 10.2196/47288 (PMC10594134; doi:10.2196/47288)
Supplement: Multimedia Appendix 2 [file humanfactors_v10i1e47288_app2.docx]

Multimedia Appendix 2

Search strategy for MEDLINE and CINAHL.

|  | 1. **Indigenous** | 1. **Immigrant, refugee** | 1. **Canada** | 1. **Virtual health** |
| --- | --- | --- | --- | --- |
| **MEDLINE** | - Indigenous Peoples - Indians, North American+ - Health services, Indigenous | - Emigrants and Immigrants+ - Transients and Migrants - Undocumented Immigrants - Emigration and Immigration - Health Disparity, Minority and Vulnerable Populations - Refugees - Ethnic and Racial Minorities | - Canada+ | - Telemedicine+ - Teleradiology - Telerehabilitation - Telepathology - Remote consultation+ - Cell phone+ - Cell phone use - Telecommunications+ - Internet of things - Internet-based intervention - Call centers - Hotlines - Wearable electronic devices+ - Computers, Handheld - Biosensing techniques+ - Remote sensing technology |
| **CINAHL** | - Aboriginal Canadians+ - Indigenous Health - Indigenous Peoples+ - Health services, Indigenous - First Nations of Canada |  |  | - Cellular phone+ - Telemedicine+ - Telerehabilitation - Telepsychiatry - Telehealth+ - Telepathology - Teleradiology - Internet of things - Internet-based intervention - Telephone information services - Wearable sensors+ - Digital technology+ - Mobile applications - Computers, hand-held+ - Biosensing techniques+ |
| **Keywords** | - Indigenous* - Aboriginal* - First nation* - Inuit* - Metis | - Refugee - Migrant* - Immigrant* - emigrant* - Newcomer* - Resettle - Displaced person - Displaced population - Foreigner* - Ethnic - Minority | - Canada - Canadian - Alberta - Ontario - British Columbia - Saskatchewan - Manitoba - Quebec - New Brunswick - Nova Scotia - Prince Edward Island - Newfoundland - Nunavut - Yukon - Northwest Territories | - Healthcare tehcnolog* - Health care tehcnolog* - Health technolog* - Communication technolog* - Wireless technolog* - Cell phone* - Cellular phone* - Smartphone* - Mobile phone* - Tablet - Computer - Telephone - Text messag* - Email - Mobile app - Helpline - Constant health monitor* - Remote health monitor* - Home health monitor* - Mobile health montir* - Remote patient monitor* - RPM - Remote sensing - Biosensor - Wearable sensor - Medical sensor - Remote tracking - Telemedicine - Telecare - Telehealth - Telecommunicat* - Teleconferenc* - Teleconsult* - Telenursing - Telemonitor - Telehome - Teletherap* - Telerehab* - Electronic health* - Virtual health* - Virtual consult* - Virtual therap* - Virtual care - Digital health* - Digital consult* - Digital therap* - Digital care - Remote health* - Remote therap* - Remote monitor* - Remote care - Video conferenc* - eHealth - e-Health - mHealth - m-Health - e-visit - e-medicine - e-rehab* |
